# Personal Health Train Architecture with Dynamic Cloud Staging

**DOI:** 10.1007/s42979-022-01422-4

**Published:** 2022-10-17

**Authors:** Luiz Olavo Bonino da Silva Santos, Luís Ferreira Pires, Virginia Graciano Martinez, João Luiz Rebelo Moreira, Renata Silva Souza Guizzardi

**Affiliations:** 1 Faculty of Electrical Engineering, Mathematics and Computer Science, University of Twente, PO Box 217, Enschede, 7500 AE The Netherlands; 2 Leiden University Medical Center, PO Box 9600, Leiden, 2300 RC The Netherlands

**Keywords:** Personal health train, Cloud, Staging station, Data station, Privacy preservation

## Abstract

Scientific advances, especially in the healthcare domain, can be accelerated by making data available for analysis. However, in traditional data analysis systems, data need to be moved to a central processing unit that performs analyses, which may be undesirable, e.g. due to privacy regulations in case these data contain personal information. This paper discusses the Personal Health Train (PHT) approach in which data processing is brought to the (personal health) data rather than the other way around, allowing (private) data accessed to be controlled, and to observe ethical and legal concerns. This paper introduces the PHT architecture and discusses the data staging solution that allows processing to be delegated to components spawned in a private cloud environment in case the (health) organisation hosting the data has limited resources to execute the required processing. This paper shows the feasibility and suitability of the solution with a relatively simple, yet representative, case study of data analysis of Covid-19 infections, which is performed by components that are created on demand and run in the Amazon Web Services platform. This paper also shows that the performance of our solution is acceptable, and that our solution is scalable. This paper demonstrates that the PHT approach enables data analysis with controlled access, preserving privacy and complying with regulations such as GDPR, while the solution is deployed in a private cloud environment.

## Introduction

In the last decades, the progressive spread of information technologies in the global society has caused a substantial increase in the amount of generated data [1]. These data are of paramount value for the modern way of life and spread through all domains, from science to life style to commerce and to security. In the healthcare domain, for instance, these data can foster medical advances and improve healthcare services, improve disease surveillance, and enable clinical decision support and population health management, to mention just a few benefits [2].

Until recently, data analysis required data to be copied and moved to a central location where they would be eventually combined with data from other sources. This approach requires potentially large amounts of data to be moved around, so that centralising them is in general not convenient anymore. From technical and economical perspectives, it is increasingly unlikely that a single organisation or individual can afford to collect and store all the needed data and maintain their required infrastructure. Another argument against data centralisation comes from a social perspective related to the ethical and legal restrictions to the sharing of privacy-sensitive data. Regulations such as the European General Data Protection Rules (GDPR) defines rules to protect the access of personal data and have an impact on the way data can be stored and processed [3]. Therefore, to comply with these regulations and, at the same time, harness the potential of the massive amount of data available, a distributed and privacy-preserving data analysis approach is necessary.

Distributed learning allows data from different source locations to be analysed without the need to move them to a central location [4]. In the healthcare domain, the *Personal Health Train* (PHT) is an approach that have been gaining momentum in the last years. The Personal Health Train initiative started in 2016 when a number of life sciences researchers in the Netherlands discussed the idea of a decentralised and privacy-preserving data and services infrastructure that could facilitate the reuse of data. This infrastructure should be based on the FAIR principles [5] to guarantee that not only the data but all relevant elements of the infrastructure are findable, accessible, interoperable and reusable. To introduce the initial ideas in an intuitive way, the Dutch Techcenter for Life Sciences produced an animation video^1^ in collaboration with the involved researchers, showing the main elements of the intended infrastructure and illustrating some of the expected use cases. The main idea of the PHT is that the algorithms move to the data instead of the other way around. The PHT uses the analogy of a train system, in which *trains* (the analysis algorithm) move through (data) *stations*, so that in each station data can be accessed and analysed. This approach allows analysis to be performed on scattered data, including sensitive data, without the data leaving their organisational boundaries, so that data privacy and control can be preserved, and ethical and legal concerns are observed [6].

The *personal* part of the Personal Health Train is about giving to the data controller the means to exercise controlling authority over their personal data that are hosted and managed in different locations. This allows data controllers to more precisely know where their data are located and also to determine the data access and reuse conditions. The PHT supports dynamic consent, in which the data controller is asked to explicitly consent for a data requester to access his/her data under some specific context. Regarding privacy and security, the main benefit of the PHT is that data processing happens within the administrative realm of the data controller. In this way, data analysts are able to get valuable information from different sources, including sensitive data, without directly accessing the data [7].

Data analysis algorithms require input data. Although seemly obvious, this statement embeds a number of assumptions such as that the data have been discovered, are accessible, match the algorithm’s requirements and are allowed to be used. In biomedical research, there is an increase adoption of machine learning (ML) techniques for identification, classification and prediction of, for instance, pathologies and their outcomes [8–11]. A commonality among these works is that ML algorithms require an often large amount of training data to improve their results. By following the FAIR principles, the PHT approach improves the findability, accessibility, interoperability and reusability of the data made available in the Data Stations. This is expected to improve the availability of potential training sets for these algorithms.

The PHT vision foresees data stations of different sizes and capabilities. For instance, large hospitals would have large stations containing data from a significant number of patients, while small medical practices would have a station containing only the data of its patients. Since computing capacity is necessary whenever processing is expected to be performed, a suitable environment should be available in the organisation hosting the data station. However, the IT infrastructure of many organisations may not be powerful enough to support the processing required by arriving trains in addition to their own operational processes. In this case, a mechanism has to be devised to allow more powerful processing environments to be dynamically staged for executing the incoming algorithms while keeping the data under control of the data controller and sensitive data protected.

This paper reports on our efforts to extend the PHT approach to allow data to be processed in the cloud, dynamically augmenting the processing power of the IT infrastructure of healthcare organisations. Our solution not only fulfils the functional requirements of the PHT approach, but it also complies with privacy regulations, particularly the GDPR. The paper describes the design and implementation of our solution, and demonstrates its suitability with a simple yet representative case study.

This paper extends [12] by giving more details on the PHT architecture and describing the process that determines whether a data station needs to be staged. Other small adjustments have also been made in the staging process to better reflect the current status of its design and development.

This paper is further structured as follows: the next section introduces the PHT architecture, explaining its main components, the third section discusses the data visiting process that determines the data stations to which a train is forwarded, the fourth section presents the data staging process that is performed in case a train is executed in the cloud, the fifth section presents the implementation of our solution, by justifying our choices of technologies, the sixth section discusses the case study we used to validate our solution, the seventh section discusses related work and the last section gives our conclusions and recommendations for future work.

## Architecture

The Personal Health Train (PHT) architecture adopts the metaphor of a train system in which trains move around and stop at stations. In the PHT, the trains represent analysis (processing) algorithms that visit stations where data are made available for processing. The architecture is specified with the Archimate 3.1 language [13], which is the OMG standard for Enterprise Architecture modelling as a visual language with a default iconography for describing, analysing, and communicating architectural concerns. Parts of the PHT architecture are also specified with UML [14], such as sequence and activity diagrams for describing behavioural aspects.

### Components and Roles

**Fig. 1:**
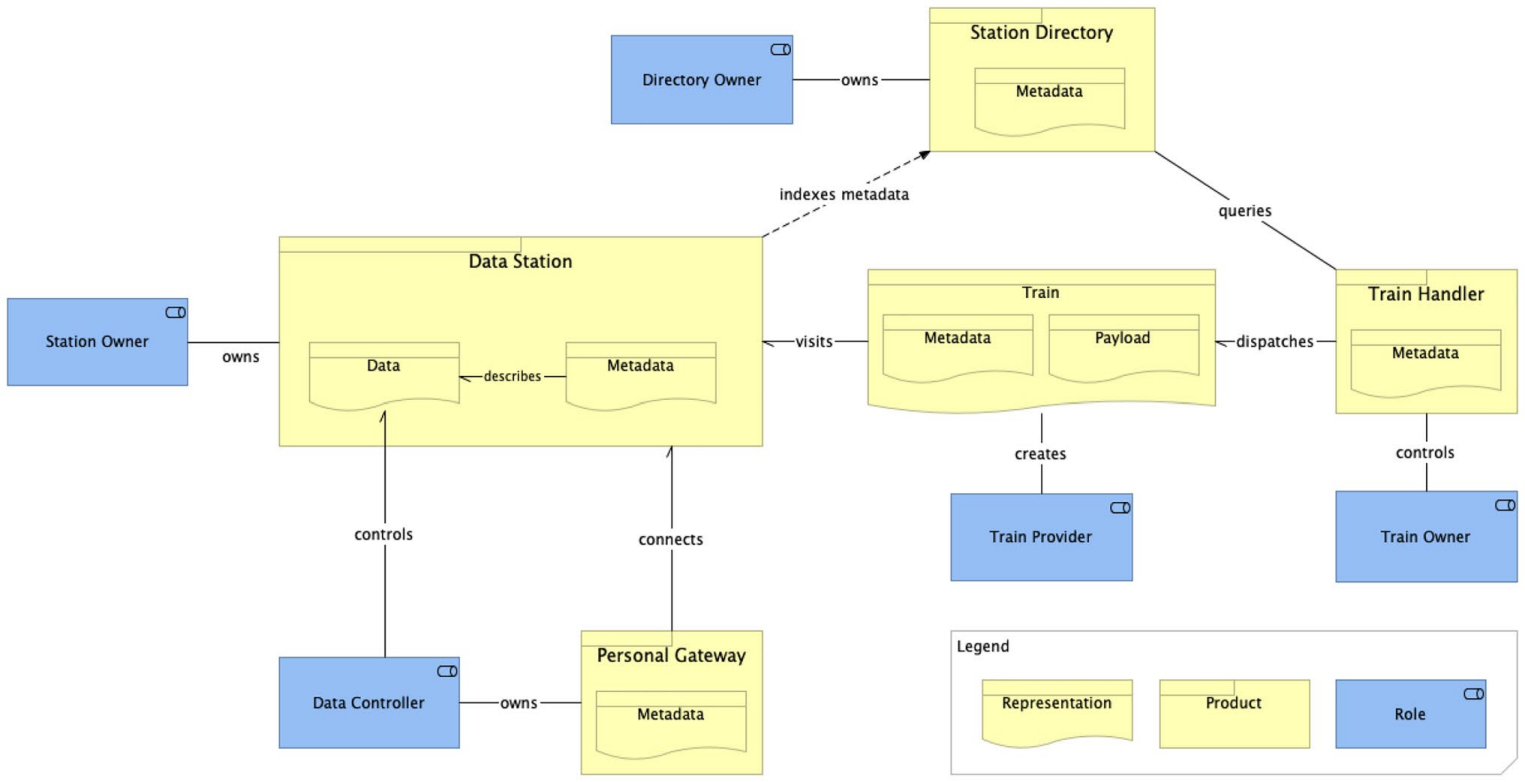
Main roles and components of the PHT architecture

Figure 1 shows the main architectural components of the PHT, namely:

- **Data Station** is a software application responsible for making data and their related metadata available to users under the accessibility conditions determined by applicable regulations and the related Data Controllers.
- **Personal Gateway** is a software application responsible for mediating the communication between Data Stations and Data Controllers. The Data Controllers are able to exercise their control over the data available in different Data Stations through the Personal Gateway.
- **Station Directory** is a software application responsible for indexing metadata from the reachable Data Stations, allowing users to search for data available in those stations.
- **Train** represents the way data consumers interact with the data available in the Data Stations. Trains represent a particular data access request and, therefore, each train carries information about who is responsible for the request, the required data, what will be done with the data, what it expects from the station, etc.
- **Train Handler** is a software application that interacts with the Stations Directory on behalf of a client to discover the availability and location of data and sends Trains to Data Stations.

Related to the components above, the following roles played by stakeholder also depicted in Fig. 1 have been identified:

- **Station Owner** is the role of being responsible for the operation of a Data Station.
- **Directory Owner** is the role of being responsible for the operation of a Station Directory.
- **Data Controller** is the role of controlling rights over data.
- **Train Provider** is the role of being responsible for the creation of a specific Train, e.g. the developer of a specific analysis algorithm.
- **Train Owner** is the role of using a Train Handler to send Trains to Data Stations.

Since the PHT data analysis is mainly performed at the data source side, appropriate definitions are necessary to determine where to find, how to access, how to interpret and how to reuse the data. These definitions are provided using appropriate metadata to describe each of the PHT elements as depicted in Fig. 1. Therefore, the PHT infrastructure relies on the FAIR (Findable, Accessible, Interoperable, Reusable) data principles [5], which should apply to all elements of the architecture, including the Train and Data Station, focussing on the reusability of distributed data with distributed analytics.

### Train Types and Structure

**Fig. 2:**
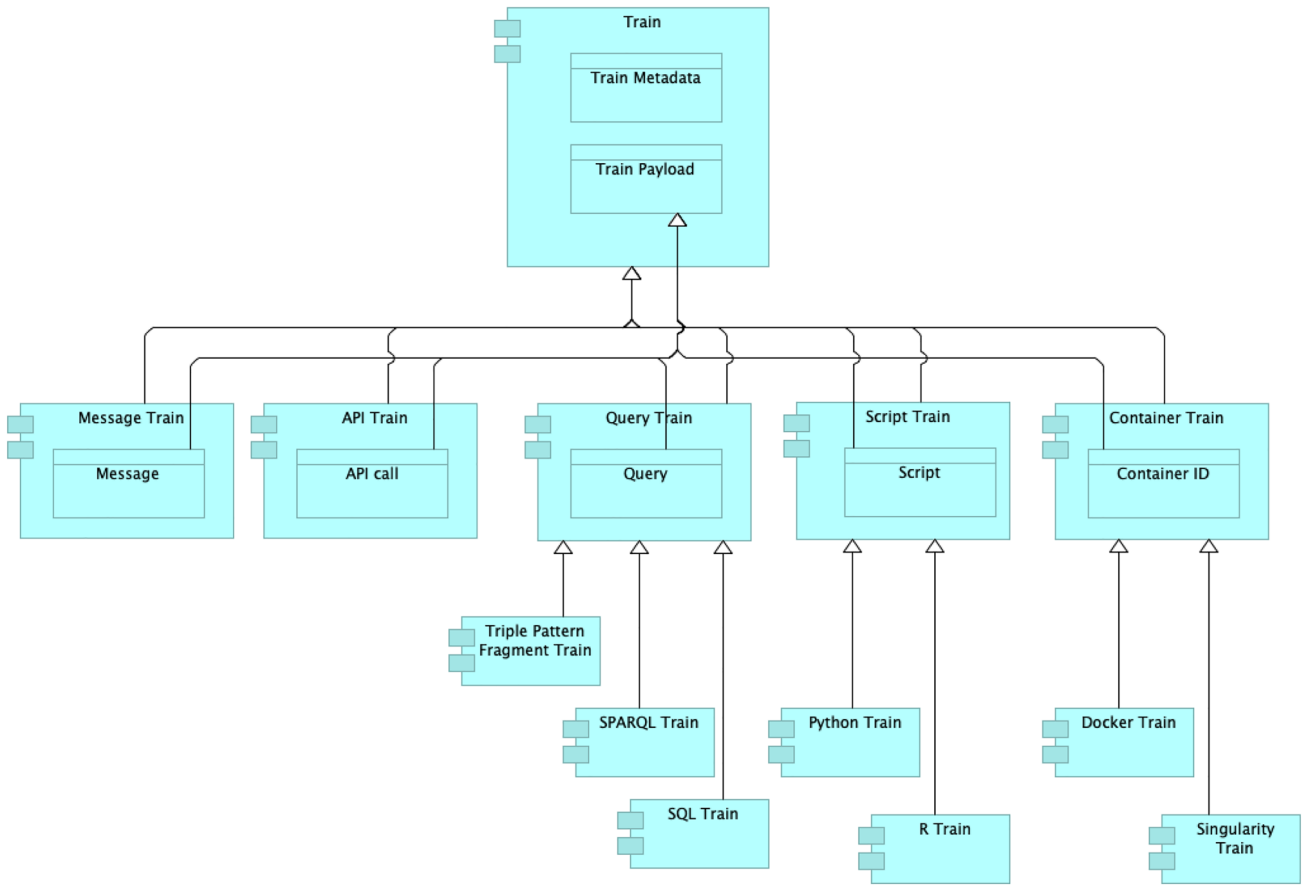
Examples of Train types in the PHT approach

Trains represent algorithms that manipulate data. We can use different methods to realise data manipulation such as queries, API calls, container technologies, scripts, among others. Therefore, Trains can have different types, according to the data manipulation method they use. Figure 2 depicts examples of Train types as application components with their respective data objects.

A Train is composed of two parts (see Fig. 2), namely the Train metadata and its payload. The metadata provides information about the Train, including its creator (Train Provider), its dispatcher (Train Dispatcher), the required input data, the expected output, what is supposed to happen with the data (the process), its purpose and its computation requirements. Most of the Train metadata properties are applicable to any type of train. In contrast, the train payload depends on the train type. For instance, in a SPARQL train, which is a specialisation of Query Train, the payload is the SPARQL query, while in a Docker train, which is a Container Train, the payload is the identifier of the Docker image stored in a Docker repository.

### Data Station Services

**Fig. 3:**
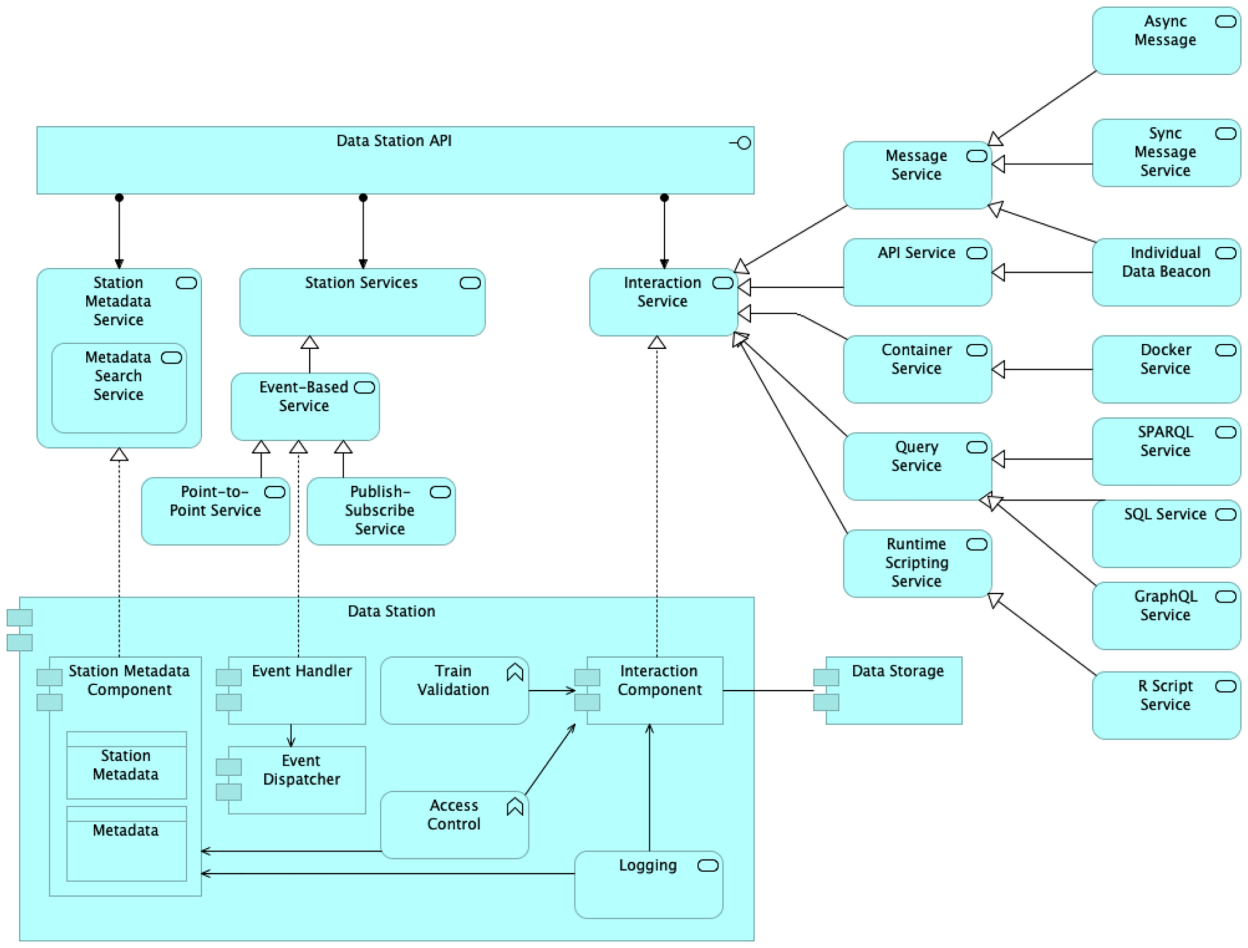
Data Station functions, services, interfaces and internal components

Data Stations provide access to their functionality through their API. In the PHT architecture, we classified the services offered by the Data Stations in three groups, namely Station Metadata Service, Station Services and Interaction Service, as depicted in Fig. 3.

The Station Metadata Service is responsible for managing and providing access to the metadata of the Data Station itself and its contents. This includes interfaces for managers and data stewards to add and edit metadata records, and for users and client applications to search and retrieve the available metadata content.

The Station Services include Data Station-specific functions, such as the connection between the Data Station and the Personal Gateway, subscription services where, for instance, the Station Directory can subscribe to received notifications on updates on the metadata content of the station.

The Interaction Service is responsible for receiving and executing trains. Since we have different types of trains, stations need to specialise this service with the specific functionality required to support each of the train types. Station owners may restrict the types of interaction services a given station supports.

## Data Visiting Process

The PHT approach encourages and regulates data reuse since Trains reaching the Data Stations access the necessary data and complete their tasks without giving direct data access to their end-users. In the PHT, a data consumer who wants to analyse or manipulate data in any way, plays the role of a Train Owner and uses the Train Handler (client application) to dispatch a Train to the relevant Data Station.

Figure 3 shows how the Interaction Component of the Data Station executes the train payload. This component serves as an intermediary between the data source (Data Storage in Fig. 3) and the Train. Therefore, data manipulation (analysis, copy, creation, etc.) occurs at the Data Station and under the supervision of the Station Owner. This allows, for instance, that the station has the opportunity to inspect the output of the train execution and check whether the results are allowed to be returned to the Train Owner.

**Fig. 4:**
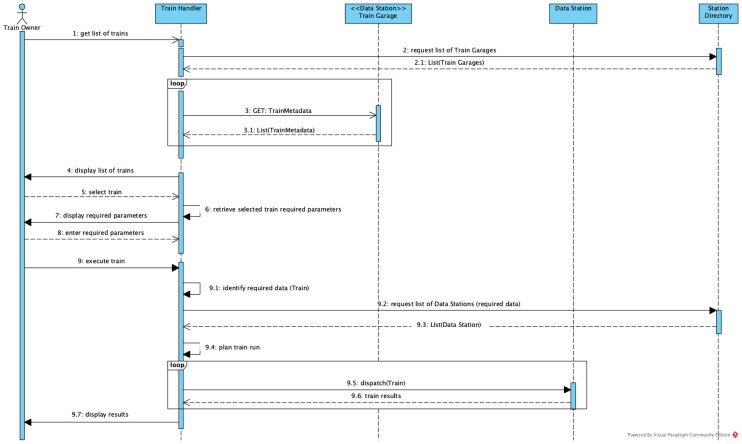
Sequence of train dispatching interactions

Figure 4 depicts the sequence of interactions involved in selecting and dispatching a train. The process starts with the Train Owner using its Train Handler application to select a train. In order to list the available trains to its user, the Train Handler interacts with the Station Directory to request the list of Train Garages, which are Data Stations specialised in providing Trains instead of or in addition to data. With this list, the Train Handler invokes each Train Garage to retrieve the metadata of the available trains. These options are then returned to the Train Owner, who selects a train to be dispatched. From the train metadata, the Train Handler retrieves the required parameters of the train. For example, in a health application one parameter could indicate the disease the train provides analysis for. The parameters are provided by the Train Owner, who then proceeds to dispatch the train. The Train Handler identifies the required input data from the train metadata, searches in the Station Directory for the Data Stations that provide the required data, plans the train execution and dispatches the train to the discovered stations. Once the train is executed, the Train Handler receives the results and presents them to the Train Owner.

## Data Staging

**Fig. 5:**
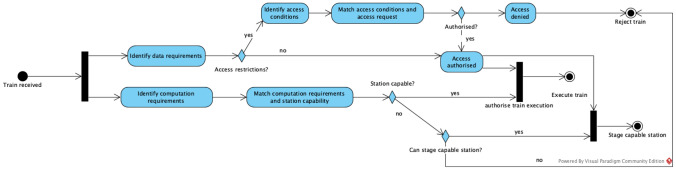
High-level steps of the train evaluation process

An important step of the data visiting process that is performed by a Data Station is the decision to accept or refuse the execution of the train. To make this decision, the station needs to consider a number of different variables such as who sent the train, for which purpose, the required data, the expected results and the computation requirements of the train. Figure 5 gives the high-level steps of the train evaluation process. In this process, once a train gets to the station, two parallel evaluations are triggered, one to evaluate the train data access requirements and another to evaluate the train computation needs. In this process we have three possible outcomes, (i) the train is executed because both computation and data access requirements are matched, (ii) the train is rejected because either the data access has been denied or the station does not have enough resources to run the train, or (iii) the data access has been authorised, the station is not capable of executing the train but it can stage a capable station with enough resources to run the train.

The train evaluation process path related to data can become significantly complex as issues such as privacy, security, access control and data structure and semantics should be taken into consideration. We are progressively working on these issues by investigating the use of semantic descriptions of data requirements and data offerings to support automated matching. However, in this paper, we focus on the situation in which (i) the original station has asserted that the incoming train has access to the required data, and (ii) the station is not able to execute the train but has determined the feasibility of dynamically deploying another station with the required data in an environment with enough computing resources, such as a cloud platform. The architectural design of the Data Station supports that the Data Station platform runs the Train either locally or uses a Staging Station in the cloud, depending on the computing resources available at the main Data Station and the Train requirements. This capability improves flexibility and scalability, using local resources or extending the infrastructure resources with the Staging Station when required.

**Fig. 6:**
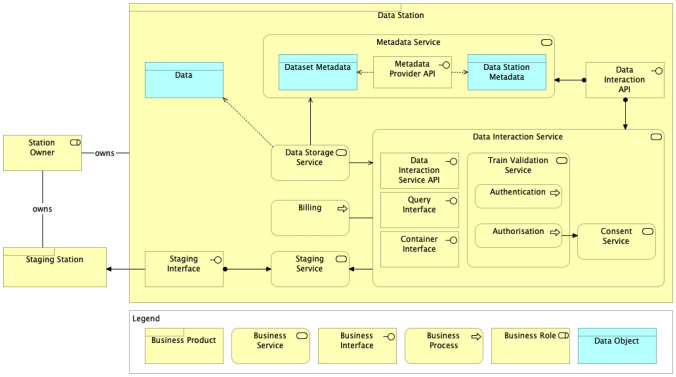
Data Station architecture with staging components

Figure 6 depicts the proposed Data Station architecture with the elements related to staging. The Data Interaction Service provides functionality that allows Trains to access the data available at the Data Station and also validates the incoming Train via the Train Validation Service, as depicted in Fig. 5. The Data Interaction Service assesses whether the Train behaves according to the Station requirements and the Train description provided in the Train metadata. Whenever the data required by a Train have access restrictions, the Data Interaction Service also enforces the required access control through authentication and authorisation. In some cases, the authorisation process triggers the Consent Service, which is responsible for requesting a consent from the responsible person or entity to grant access to the data.

The Data Station Metadata Service provides access to the metadata of the Data Station and of all data sets made available through this Station. External applications willing to retrieve metadata from the Data Station invoke this metadata service to accomplish the task.

Once the station has determined that it needs to stage a new station in the cloud in order to execute the train, the Staging Service proceeds to deploy a new Data Station in the cloud through its related Staging Interface, configuring the environment with the computing capabilities compatible with the Train requirements. The Staging Service uses the Data Interaction Service to retrieve the data required by the Train and copy them to the newly deployed Staging Station. Finally, the Train is forwarded to the Staging Station where it is expected to be executed.

The Station Owner of the original Data Station is also responsible for the Staging Station. The staging process should therefore be transparent to the Train Owner. However, the use of an external platform can incur an extra cost. For this reason, we defined a Billing component, which can be used at the Station Owner’s convenience and can accrue the staging costs that should be covered by the Train Owner, depending on prior agreements.

The Staging Station is an extension of the regular Data Station and it behaves like the original Data Station, but with some additional features.

**Fig. 7:**
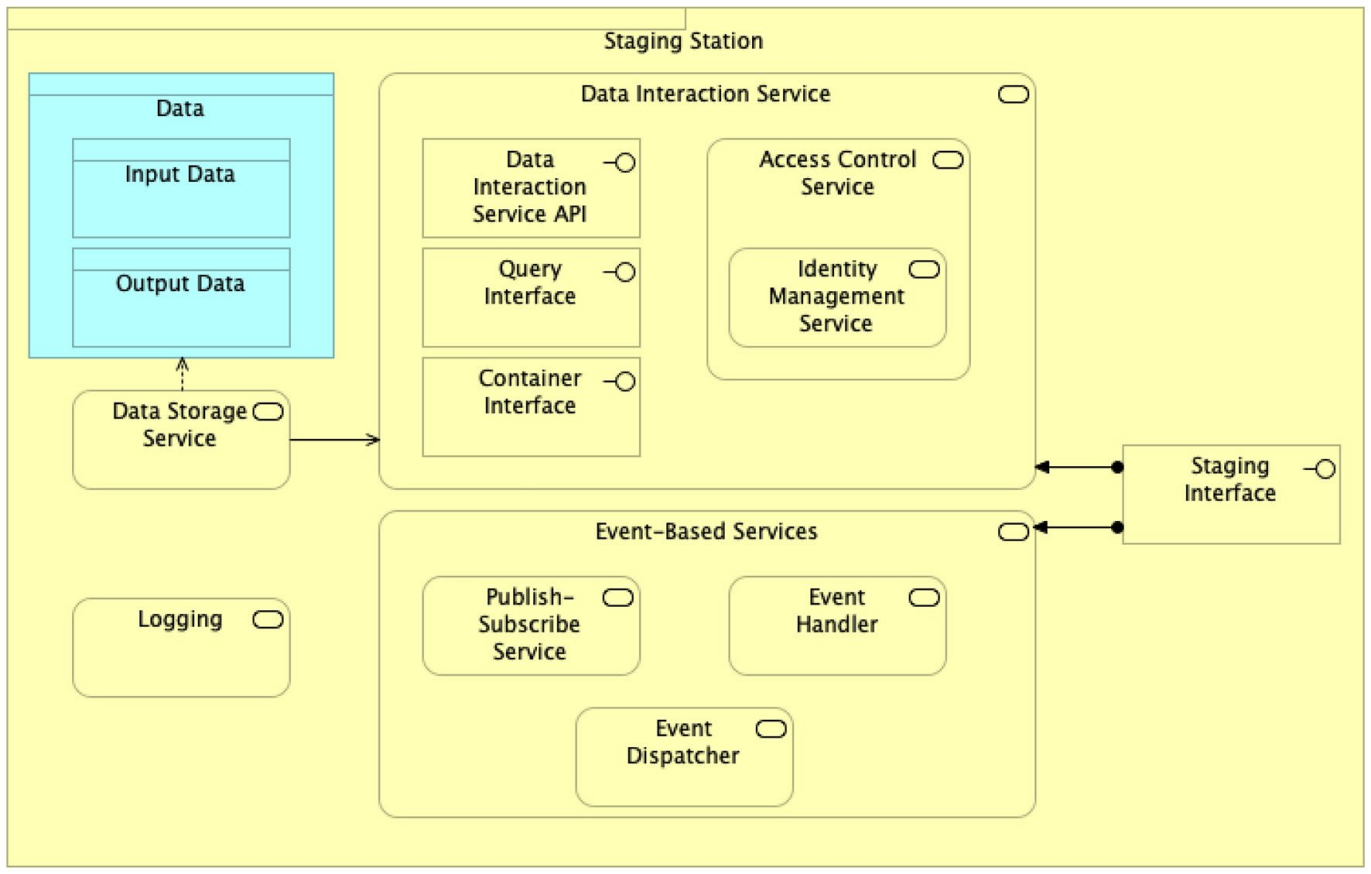
Staging Station architecture

Figure 7 depicts the architecture elements of the Staging Station, which offers the following services:

- **Access Control Service** offers access control to the cloud environment, but only to the Data Station Owner. If needed, more users can be added and get specific permissions and policies to execute particular tasks. Communication between the components in the cloud is denied by default to provide a proper secure environment. The Identity Management Service can later provide roles to allow or deny access to the other resources deployed and used by the Staging Data Station, such as storage and computing instances.
- **Data Storage Service** stores the input and output data. The input data are selected at the original Data Station based on the Train needs and moved to the Staging Data Station in the cloud. The output data result from the Train execution, and are sent to the original Data Station.
- **Even-Based Services** The Staging Platform provides event-based services to automate the execution steps. For instance, when the data are entirely moved to the cloud, the Staging Data Station notifies the cloud computing instance in which the Train can be executed. Further, the original Data Station may subscribe to be notified when the Train execution finishes, to harvest the output data once they are available. Events and trigger actions are achieved through the Event Handler and Event Dispatcher services, which listens to the events issued by infrastructure components to create rules and trigger actions, and executes the actions provided by the Event Handler, respectively.
- **Logging Service** logs the data access interactions, enabling regulatory compliance and security, but also operational tasks. It identifies which actions were taken by whom, the resources that were acted upon, who accessed which data when the event occurred, and other details to help analyse and respond to an activity. This is a requirement for GDPR compliance, but it is also used to communicate with the Event-based services to launch tasks when an event occurs.

## Solution Implementation

In this section, we present our implementation of the proposed Staging Station architecture that has been developed to process a Container Train, which is a Train that represents a Docker image. We begin by discussing the selection of tools, followed by the implementation in the dynamic infrastructure platform.

### Technologies

For the dynamic platform to stage Data Stations, we chose Amazon Web Services (AWS), due to its GDPR compliance [15], free-tier resources for testing, plenty of options for infrastructure resources [16] and its global infrastructure, with multiple locations worldwide and especially in Europe. For the provisioning tool, we chose Terraform^2^, since it is open source, supports multiple dynamic platforms and has declarative configuration. Furthermore, most alternatives are vendor-specific solutions and could create yet another vendor lock-in. The extensive integration and support offered by Terraform confirmed our choice for AWS as dynamic platform.

Terraform is convenient because it allows many infrastructure components to be implemented through pieces of code that can be deployed at the same time. Terraform provisions the resources of a dynamic platform, and a Terraform provider is used to interact with the APIs and expose the resources from the corresponding dynamic platform. In our implementation, the AWS provider is used for provisioning all the required resources. Besides, we chose the closest European AWS region (Frankfurt) in order to comply with GDPR. Terraform has been installed in the machine that runs the Data Station.

The implementation comprises two parts: the Data Station, which runs in a laptop, and the Staging Station, which runs in AWS. Since we implemented our data staging prototype to run Container Trains, once the Train is set up to run in a Data Station, the Station takes the container identifier from the Train payload and retrieves the container image from its Train Registry. In our prototype, we used the Amazon Elastic Container Registry (ECR) service, which supports Docker images, as the Image Repository.

We implemented the Data Station in our prototype on a computer with 1.8 GHz Dual-Core Intel Core i5 and 8 GB memory. We implemented an API that plays the Data Station role and interacts with the Staging Station. In addition to Terraform, the technologies used in the implementation and installed in the computer are Docker client, AWS SDK for Python, NodeJS, and Express. The Data Station API is configured in NodeJS and exposed to the Internet via the *localtunnel npm* tool.

Our implementation supports the functionality triggered after the decision that the Train cannot run at the original Data Station due to lack of enough computing resources. In addition, it assumes that the Train can have access to the required data.

### Interactions

**Fig. 8:**
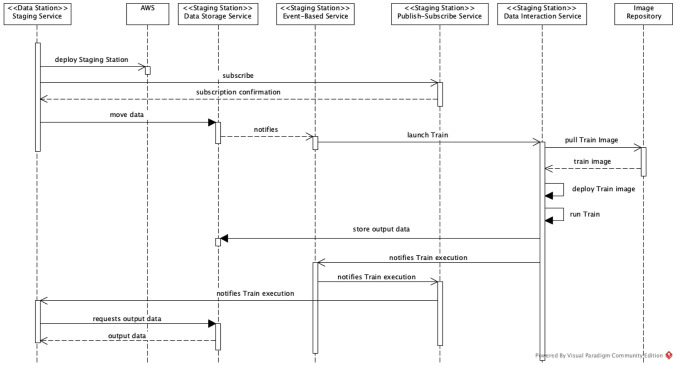
Interaction sequence for Staging Station

Figure 8 depicts the sequence diagram that shows the interactions between our implementation components to support the deployment and execution of the Train in the Staging Station. Once the decision to stage a new station in the cloud has been made, the Data Station Staging Interface launches the Staging Station in AWS as described in the Terraform definition files. This allows all the necessary components to be provisioned in the AWS cloud at the same time.

Once the Staging Station is deployed, the (original) Data Station subscribes to the Staging Station Publish-Subscribe Service to receive a notification when the Train execution is completed. The Data Station also moves the required data to the Staging Station through its Data Storage Service. Once the required data have been transferred to the Staging Data Station, the Event-Based Service immediately triggers the Data Interaction Service to launch the Train. In order to do so, the Data Interaction Service pulls the Train Image from the Image Repository, deploys it in the Staging Station and executes it. Once the Train execution is completed, the resulting data are stored by the Data Storage Service.

When the Train finishes its execution, the Data Interaction Service informs this via the Event-Based Service to the Publish-Subscribe service. Finally, the Publish-Subscribe Service sends a notification message to the Data Station, which can proceed to transfer the output data from the Stating Station. At this point if necessary, the Data Station can verify whether the output data complies with the expected output as defined in the train metadata. Once all checks have been done, the Data Station sends the output data to the requesting Train Handler.

### AWS Services

**Fig. 9:**
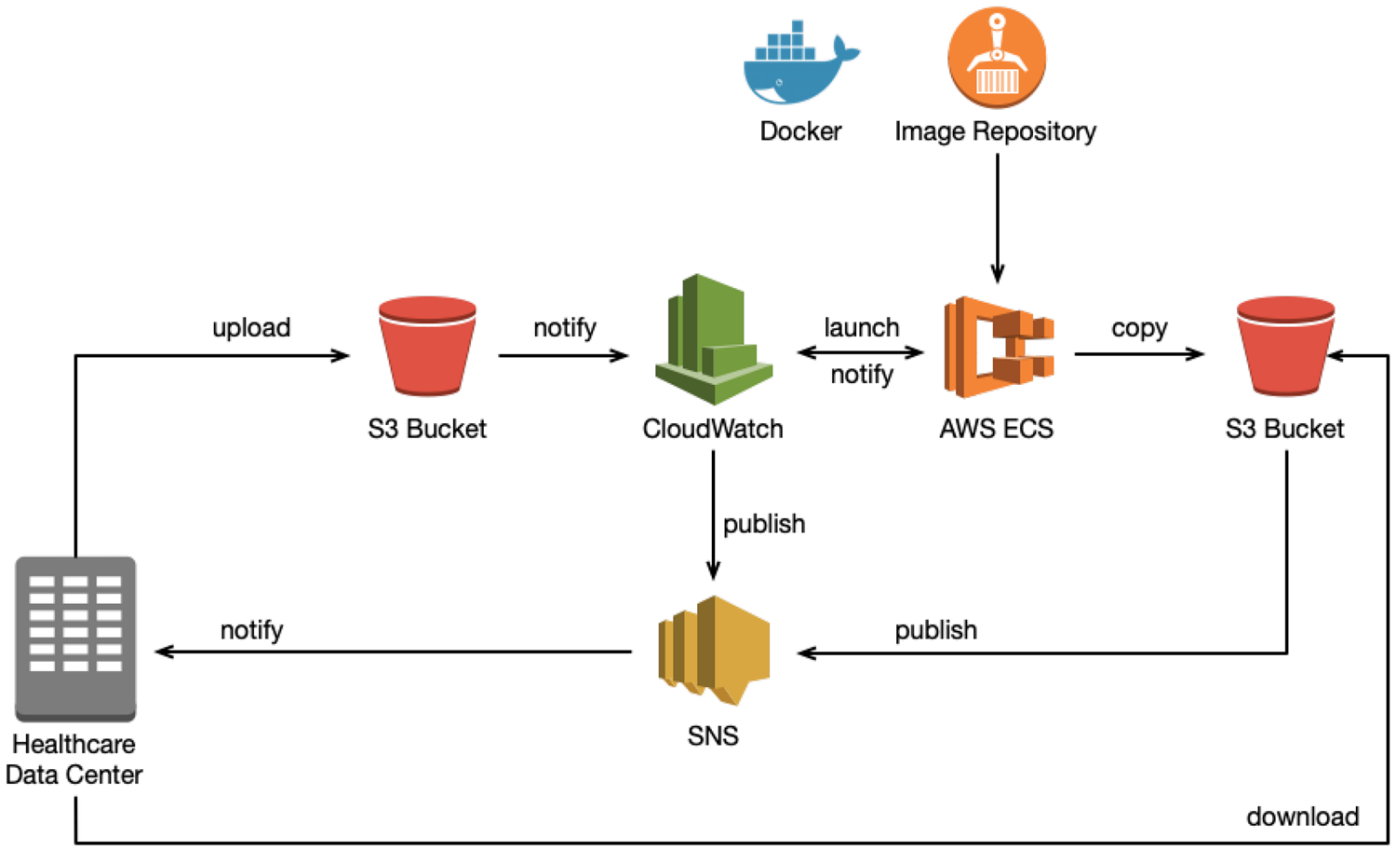
Implementation in AWS

Figure 9 illustrates the Staging Data Station implementation we deployed in the AWS cloud in accordance with the interaction diagram of Fig. 8.

Table 1 shows the AWS services we used in our implementation, as well as the service that implements each PHT component from Fig. 7.

**Table 1:** PHT components and AWS services

PHT component	AWS service	Description
Data storage service	Simple storage service (S3)	Provides object storage through a web service interface
Event handler, event dispatcher	Cloud watch	Monitoring service that provides data and actionable insights for AWS infrastructure resources
Publish-subscribe service	Simple notification service (SNS)	Using SNS topics, publisher systems can fan out messages to many subscriber systems, including HTTP endpoints
Container environment	Elastic container service (ECS) fargate	Computation runtime environment based on serverless technology that facilitates deployment, so that we do not need to be concerned about how many resources assign in advance
Access control	Identity and access management (IAM)	Manages access to AWS services and resources securely
Networking	Virtual private cloud (VPC)	Creates a custom networking environment
Image repository	Elastic container registry (ECR)	Fully managed Docker container registry

#### Authentication

In order to create an environment in AWS, we first need an Amazon Web Service Account and a special authentication method. We used Multi Factor Authentication (MFA) to access the AWS console. We assigned an Admin role to the Station Owner, having a name and two keys, namely, the public assess key and the secret key. In this way, the desired connection to the environment is established in an absolutely reliable and secure way.

The authentication credential are configured in the Data Station, allowing it to interact with AWS through its Staging Service to trigger the deployment of a Staging Station. The creation of the AWS account and the configuration of the Data Station with the AWS account credentials are manual steps that have to be taken by the Station Owner.

#### Publish-Subscribe Service

We use Amazon SNS to implement the Publish-Subscribe Service. The SNS can respond with three types of messages: (i) subscription confirmation, (ii) confirmation to unsubscribe, and (iii) the actual notification. The messages sent by SNS use HTTP POST requests with the message type on the header, allowing us to identify the type of message and run specific jobs in the Data Station Staging Interface, as shown in Fig. 8.

#### Storage

The Data Storage Service is implemented with Amazon S3, so from now on it is referred to as the *bucket*, in accordance with the AWS S3 terminology. We used three buckets by design, to store input data, output data and log files, respectively. The use of several buckets provides more granular security and facilitates automation, triggering different actions on each of them: the availability of input data triggers the Train execution, while the availability of output data triggers the download and smooth data retrieval at the end of the Train execution. Table 2 shows our buckets and their use.

**Table 2:** Buckets description

Bucket	Use
Input data	Stores the data required by the Train transferred from the original Data Station
Log	Stores event history logs of the AWS account activity in the region, besides helping the event-based service launch other resources dynamically
Output	Stores the results of the Train execution. The original Data Station retrieves the data from this bucket at the end of the Train execution

The implementation supports client-side encryption and server-side encryption for protecting data stored in the cloud and in transit against unauthentic and unauthorised access, while ensuring that they remain intact and available. Client-side encryption is used to protect data in transit by encrypting data before sending them to AWS S3. HTTPS is used to guarantee secure connections. If the healthcare organisation has a Virtual Private Network (VPN) infrastructure, we recommend to establish a private connection to the cloud. For the server-side encryption, a unique encryption key is generated for each object, and data are encrypted using the 256-bit Advanced Encryption Standard 256 (AES-256). After that, a securely stored and regularly rotated master key encrypts the encryption key itself. Users can choose between mutually exclusive possibilities to manage the encryption keys. The input and output buckets use unique Amazon S3-Managed Keys (SSE-S3) with strong multifactor encryption.

#### Data Transfer

Data transfer is configured with the Terraform files. We used the *depends on* meta-argument provided by Terraform to express dependencies between components. In this case, data are transferred once the remaining resources are created in the cloud, so that we can move data without concerns. Furthermore, we verify the integrity of the uploaded data with Message Digest (MD5) checksum in order to detect data corruption.

#### Event-Based Services

Usually, AWS Services generate notifications when an event occurs, and these events are used to trigger an action. However, these actions have to be stored somewhere, and rules and targets should be defined based on them, so we use Log Bucket to store all the input bucket actions. We create a CloudTrail that reports the activities of objects in the input bucket, which are seen as events by the CloudWatch service. After that, we configure an upload S3 event rule in CloudWatch. Once a rule condition is fulfilled, the CloudWatch target triggers an action. Accordingly, when data are completely uploaded to the Input Bucket, our system launches a compute engine for containers using ECS, and in this way the Train is executed.

#### Computing Resources

Amazon ECS makes it easy to launch containers and scale rapidly to meet changing demands, but one of the challenges during execution is the provision and management of computing and memory resources. There are several mechanisms to predict the resources required and scale them appropriately. However, the Staging Station is a temporary deployment that has the main task of providing the appropriate computing resources for the Train. ECS Fargate is a serverless solution that allocates the required amount of computing capabilities, so that instances do not have to be chosen in advance since it scales cluster capacity as required by the application.

We use an entity called a task definition to describe to ECS how to run the container. The ECS task definition can be thought of as a prototype for running an actual task, and allows for one or more containers to be specified. In our implementation, each Train is mapped onto one task definition, which describes that the Train should be pulled from the Image Repository when the CloudWatch rule matches the uploading event. Unlike Virtual Machines in the cloud, ECS Fargate is charged by vCPU and memory, and not by the hour.

#### Security

In addition to the use of authentication and encryption mechanisms, an appropriate strategy for increasing security is to classify, split, and divide everything, using roles, permissions, regions, networks or firewalls. In a cloud environment, we can implement security at different levels. In our solution, we created a Virtual Private Cloud to isolate our components from other customers in AWS. However, the resources cannot interact with each other if we do not configure a policy to allow them to interact. Therefore, we provide security via an Identity and Access Management service with access control through user definitions, roles and permissions to users in each step of the workflow. For instance, the ECS Cluster has read access to the S3 input bucket, but it does not have write permission as it only requires to get data from it. In contrast, ECS has write-access to the S3 output bucket.

The implementation uses a collection of network access rules to limit the traffic types that can interact with a resource. This collection of rules is called a security group, and each resource can be assigned to one or more security groups. The rules in a security group control the traffic allowed to an instance. If incoming traffic does not match a rule from the group, access is denied by default.

## Case Study

We evaluated the design proposed in this research with a simple analysis of COVID-19 data spread through data sets of various sizes representing different workloads. This allowed us to evaluate the system behaviour, mainly in terms of the consumed network and computing resources. We used our implementation to build a Container Train with an algorithm to process and analyse COVID-19 patients’ information. We used data sets created in the literature [17], where the authors generated synthetic data using the open-source Synthea tool, resulting in data sets containing synthetic Electronic Health Records (EHR). The experiment aimed at calculating all matching patients diagnosed with COVID-19 and evaluate our system using 10K and 100K bundles. For the patients diagnosed with COVID-19, we got summary statistics of patients who recovered and died and the care plan of the people infected.

### Evaluation Metrics

*Performance* is the most suitable quality attribute to evaluate the architecture using dynamic analysis. We used two sets of measurements for this quality at- tribute, based on the ISO 25010 standard [18] and the validation technique presented in [19]:

- **Time Behaviour** is the degree to which the response and processing time and throughput rates of a system meet its requirements when performing its functions. For this we measured the execution times from when the GET method is invoked until the resources are deallocated.
- **Resource Utilisation** is the degree to which the amount and types of resources used by a system meet its requirements when performing its functions. For this we measured the CPU Average use and RAM average use in the cloud. Moreover, we measured network traffic in the Data Station during the execution process.

### Validation

We ran the execution of the system five times per bundle. After these executions, we got an average calculation for the analysis of the system. This prevents any data disturbance caused by isolated events from having significant effects on the results. We used the tool *iftop* on the computer that runs the Data Station to collect network traffic information. Besides, we harnessed the CloudWatch monitoring tool in AWS to get the CPU and memory utilisation.

Table 3 shows the execution time for the provision and deprovisioning process for both bundles. The provisioning process comprises the Terraform files execution, data transfer, Train routing, Train processing in the cloud, and downloading the results. The deprovision process covers only the deletion of the entire cloud resources created by Terraform. We observed that the difference between the execution time of both bundles is around 15%. Table 4 shows that this behaviour can be justified by considering the average resource utilisation.

**Table 3:** Average execution time

	10K	100K
Provision	3 min. 54 seg	4 min. 14 seg
Deprovision	17 seg	20 seg

**Table 4:** Average resource utilisation

Resource	10K	100K
Network	62 Mb	70 Mb
CPU	53.5%	85.6%
RAM	12%	16%

Table 4 shows the network traffic during the provisioning process in the Data Station. The 100K bundle consumes on average around 70 Mb while the 10K consumes around 62 Mb. The slight difference in consumption is because in the 100K bundle case the multi-upload option was used due to the size of the bundle. Multi-upload divides the bundle into several chunks consuming more networking resources but in less time. Consequently, the transmission times were different but not ten times bigger than the amount of data, like it could be expected.

Table 4 also depicts the CPU and RAM average utilisation, which are the resources consumed in the cloud. We observed that the CPU average utilisation for the 100K bundle was 85.6%, almost 30% higher than for the 10K bundle. From Tables 3 and 4, we have that the cloud processing time was very similar among both bundles, but the 100K bundle consumed more resources. In general, the average memory utilisation was low and both tests used less than 20% of the available memory. This may also indicate that we could reduce the memory allocation in the cloud configuration. The overall execution time of both bundles was also very similar with a difference of around 8% despite a tenfold size difference between the two data bundles.

We can conclude that the Data Station network and cloud computing instance play a crucial role in the performance of our system, more than the amount of data. Scalability of the computing resources is achieved in the cloud, however, the network consumption depends on the network capabilities of each healthcare organisation. If we want to increase transfer data speed, we can use multi-threading techniques, although in this case many more network resources would be consumed.

Table 5 presents the results from the analysis of the 10K data sets. With 8820 infections, 96% of the people recovered, which is a high rate, from which we concluded that COVID-19 is highly contagious but not highly fatal.

**Table 5:** Mortality rate

COVID-19	Recovered	Deceased	Ventilated
(*n* = 8820)	0.9606	0.0404	0.0325

The care plan in this data set has two values, namely ‘home isolation’ and ‘hospitalised’. Table 6 summarises the statistics of patients who recovered at home and hospitals. The hospitalisation rate is considered high for the period these data were gathered. However, still, the vast majority of people followed a ‘home isolation’ care plan, i.e. they stayed and were treated at home. Table 7 shows that the Intensive Care Unit (ICU) Admission rate was high, and almost everyone at the ICU required ventilation. The death rate for people in the ICU was also high. From these data, we can conclude that patients admitted to the ICU and who use ventilation have a high probability of dying.

**Table 6:** Care plan

Care plan	Rate
Home isolation	0.7952
Hospitalised	0.2116

**Table 7:** ICU admission rate

	Care plan	Rate
Ventilation req	0.7653	1.0
Recovered	0.3573	0.1637
Death	0.6453	0.8362

These results demonstrate that our architecture implementation is capable of running a Train to perform data analysis in the cloud. Our deployment enables analysis using privacy-sensitive data sources and successive evaluation of that analysis in a secure enclave. We could deploy the Staging Data Station, so that the Train analysed the data and got a final file with the information provided in Tables 5, 6 and 7 directly in the Data Station. This also demonstrates that the standardisation of the data structures alongside a proper architecture facilitates data analysis in any environment, particularly in a distributed environment.

## Related Work

The Personal Health Train is often related to Distributed learning, a concept first introduced by Google in 2016 [4], where distributed databases are analysed at different data sources location. In this paradigm, data source organisations control the entire execution and return just the results, without sharing information and keeping the privacy of sensitive data [6].

The inspiration for the PHT was the Euregional Computer Assisted Theragnostics project (EuroCAT)^3^, which started in September 2010. From the scope of this project, the Varian Learning Portal (VLP) has been developed by Varian Medical Systems. Varian Medical Systems is an American manufacturer of oncology software and treatments, and that is why the papers reporting the use of this technology mention only applications involving cancer [20–24].

The VLP is a cloud-based system that implements user, distributed data sources, and project management. It consists of two elements, namely a master and a learning connector. A learning connector is installed at each data source to connect the VLP master to a local source. The end-user uploads his application to the VLP web portal, which can be done in MATLAB. VLP and data sources communicate via file-based, asynchronous messaging. The iterative execution of applications and communication between them is known as a *learning run*, which can be accepted or denied by each data source.

In [22], the authors demonstrated that it is feasible to use the distributed learning approach to train a Bayesian network model on patient data coming from several medical institutions. Data were extracted from the local data sources in each hospital and then mapped to codes. Besides, in the Varian learning portal, the researchers uploaded their Bayesian network model application for learning. The Varian learning portal transmits the model application and validation results between the central location and the hospitals. In [23], the authors built a logistic regression model in MATLAB R2018 to predict post-treatment 2-year survival. The VLP connector was installed in 8 healthcare institutions. In [21], the authors used the VLP to run a study to develop a radiomic signature; the authors pointed out the preference for VLP because it offered already the essential technical overhead such as logging, messaging and Internet security.

In these proofs of concept, the authors have concentrated on the algorithms to evaluate and train the data distributed geographically. They tried to demonstrate that the results are just as accurate as when data are centralised. Therefore, they have harnessed the PHT approach using VLP technology, but they did not try to define a reference architecture. Furthermore, the VLP is a proprietary solution. Moreover, the applications are not freely reusable, and only the users of each project can see what they have done. For case studies beyond cancer, this solution may not be suitable, so that other options must be explored or developed.

DataSHIELD [25] and Vantage6 [26, 27] are two other distributed processing platforms. Both have been designed and developed in the biomedical sciences domain but DataSHIELD is also used in other domains such as social sciences. Both approaches follow a similar client-server schema where a client application sends analysis requests to potentially multiple servers where the data are accessed and the analysis occurs. Concerning the interaction mechanism, DataSHIELD is based on R while Vantage6 is based on Docker images. In June 2021, the Vantage6 team announced a partnership with DataSHIELD to allow Vantage6 users to take advantage of the extensive DataSHIELD toolset by embedding these tools in Docker images used in the Vantage6 environment [28].

After the emergence of the PHT concept, a number of research groups started working on the implementation of different aspects of the ideas and concepts depicted in the PHT animation video [29]. In [3] and [7], the authors leveraged containerisation technologies for sending applications to Data Stations, more precisely Docker containers. The former created a Train containing an FHIR query and an algorithm to calculate summary statistics, then wrapped them as a Docker image and submitted it to a private Docker registry. The latter initially used a phenotype design web client to create Docker images containing a query, the metadata and the script and then submitted them to a public Docker registry.

Some aspects set our approach apart from the aforementioned distributed learning initiatives. The first is that, to the extend of our knowledge, our approach is the only one that takes the FAIR principles as the basis for design and development. As a direct consequence, metadata are given a key role to describe the data and services in a semantically rich and machine-actionable way, supporting improvements in automation such as matching between Train requirements and Data Station capabilities. In our architecture, we offer the flexibility of using different data interaction mechanisms, not imposing one particular method such as R scripts or Docker images. Finally, our proposed architecture has been defined to lead and guide the development of existing and new applications based on common interfaces, metadata formats and schemata. The reference implementation is being development to serve as a concrete example of the realisation of the architecture and not as the only implementation option. In this way, we aim at supporting flexibility as developers may decide to just extend their current applications to comply with these common interfaces, metadata formats and schemata instead of having to replace their whole system.

## Conclusion

In this work, we started from the reference architecture of the Personal Health Train and focussed on the architectural elements and processes to enable the dynamic staging of a Data Station in the cloud in case the original Data Station does not have enough resources to perform the computation required by a given Train in the premises of an organisation. In our implementation, we employed Infrastructure as Code, APIs, and event-based systems to realise a dynamic deployment in the cloud. We implemented the architecture proposal for the dynamic staging of a station using novel technologies and a popular cloud environment (AWS). We evaluated the proposal with a dynamic analysis through a case study, analysing data sets of 10,000 patients and 100,000 patients, respectively.

Our research showed that we could seamlessly deploy a more powerful computation environment when required using the cloud and automation tools, complying with the PHT principles while providing a fitting and secure site. Although our design requires moving the data to the cloud, the data are still within the realm and control of the original Data Station, keeping the same privacy levels. Moreover, our proposal complies with the main regulation for processing personal data in the cloud to keep the information as secure and private as possible, assuming that the cloud environment does not misbehave nor has been hacked. The case study showed that the instantiation and processing times of the Staging Station depend on the network in the Data Station and the computing resources consumed in the cloud. The simulation showed similar execution times with different workloads sizes, but a significant difference in the network and computing consumption, which can cause a bottleneck in the Data Station network. We highlight that the same correlation between processing time and computing resources consumed holds for the original Data Station. This leads to the conclusion that further investigation is necessary to improve the identification of the Train computing requirements so that the dynamic staging mechanism would be able to define a better provision of cloud resources for the staged station and, therefore, achieve optimal processing time. Other parameters such as execution time constraints and costs have not been included in the current implementation but should also be taken into consideration by the improved dynamic staging mechanism.

Different types of Trains have different data and computing requirements, which affect both the volume of data to be transferred to the Staging Station and the Train execution. Therefore, we think that it may be useful to design capabilities that allow the originally targeted Data Station to inform the Train Owner in case the Train cannot be executed in the station but in a dynamically deployed Staging Station in the cloud. In this way, in time-critical situations, the Train Owner can decide beforehand whether the increase in response time and the possible additional costs are acceptable, and may choose to abort the Train execution.

The case study worked adequately with a simple aggregation algorithm, so we have evidences to conclude that our system can alleviate the IT infrastructure constraints that healthcare organisations may have to ensure the PHT execution, while respecting the principles of the PHT approach.

Future research should be performed to test our solution with other use cases, by including Machine Learning algorithms in the Train or dependent transactions, for instance, to experience idle moments waiting for input data. Other Trains types with different interaction mechanisms (e.g., APIs, queries, and messages) should be implemented and tested as extensions to our system. We also propose some future work to assess the solution developed in this research, integrating the implementation to existing proof of concepts developed by organisations that contribute to the PHT initiative. Some of these implementations already have deployed a vast majority of the PHT workflow and have elaborated more robust case studies. It would beneficial to combine these efforts and to evaluate how our solution behaves by applying other metrics like performance and execution time measured from when the end-user dispatches the Train until the results are made available.

Finally, the work reported in the paper together with efforts to implement other aspects of the PHT architecture, such as data access authorisation process and dynamic consent are being further developed and tested in a number of projects such as the C4yourself, Personal Genetic Locker and the European Joint Programme on Rare Diseases. In these projects, the PHT architecture is being applied to new and existing applications, aiming at demonstrating the convergence of approaches and interoperability improvements that can be achieved by agreeing on a common architectural specification.

## Footnotes

1 https://vimeo.com/143245835.

2 https://www.terraform.io.

3 http://www.eurocat.info/index.html.
